# Proficient trigonometrical-fitted two-derivative multistep collocation methods in predictor-corrector approach: Application to perturbed Kepler problem

**DOI:** 10.1016/j.mex.2024.103045

**Published:** 2024-11-15

**Authors:** Khai Chien Lee, Muhammad Naeim Mohd Aris, Ishak Hashim, Norazak Senu

**Affiliations:** a Department of Mathematical Sciences, Universiti Kebangsaan Malaysia, 43600 UKM Bangi, Selangor, Malaysia; b Institute for Mathematical Research, Universiti Putra Malaysia, 43400 UPM, Serdang, Malaysia; c Department of Mathematics and Statistics, Universiti Putra Malaysia, 43400 UPM, Serdang, Malaysia

**Keywords:** Trigonometrical-fitted, Two-derivative multistep collocation, Collocation, Second-order ordinary differential equations, Predictor-corrector, Trigonometrical-fitted two-derivative multistep collocation method in predictor-corrector mode

## Abstract

An efficient trigonometrical-fitted two-derivative multistep collocation (TF-TDMC) method using Legendre polynomials up to order five as the basis functions, has been developed for solving second-order ordinary differential equations with oscillatory solution effectively. Interpolation method of approximated power series and collocation technique of its second and third derivative are implemented in the construction of the methods. Two-derivative multistep collocation methods are developed in predictor and corrector form with varying collocation and interpolation points. Later, trigonometrically-fitting technique is implemented into TF-TDMC method, using the linear combination of trigonometrical functions, to produce frequency-dependent coefficients in TF-TDMC method. The stability of the TF-TDMC method, with fitted parameters, is thoroughly analyzed and has been proven to achieve zero stability. Stability polynomials and regions for predictor and corrector of TF-TDMC method are developed and plotted. In the operation of the TF-TDMC method, initial conditions and the frequency for each problem (based on the exact solutions) are identified. The frequency-dependent coefficients are then adjusted according to the identified frequency. Predictor and corrector steps are implemented to estimate and refine the values of the dependent variable and its derivative, ensuring that convergence is achieved.

A numerical experiment demonstrates that the proposed method significantly outperforms other existing methods in the literature, achieving the lowest maximum global error with moderate computational time across all step sizes for solving second-order ordinary differential equations with oscillatory solutions. Additionally, it effectively addresses real-world perturbed Kepler problems. The results include a detailed discussion and analysis of the numerical performance.

• An efficient two-derivative multistep collocation method in predictor-corrector mode with trigonometrically-fitting technique (TF-TDMC) is developed for direct solving second-order ordinary differential equations with oscillatory solution.
• TF-TDMC method has been proved to acquire zero-stability and its stability region is analyzed.
• TF-TDMC method is the best among all selected methods in solving second-order ordinary differential equations with oscillatory solution, including perturbed Kepler problem.

## Graphical abstract

**Figure:**
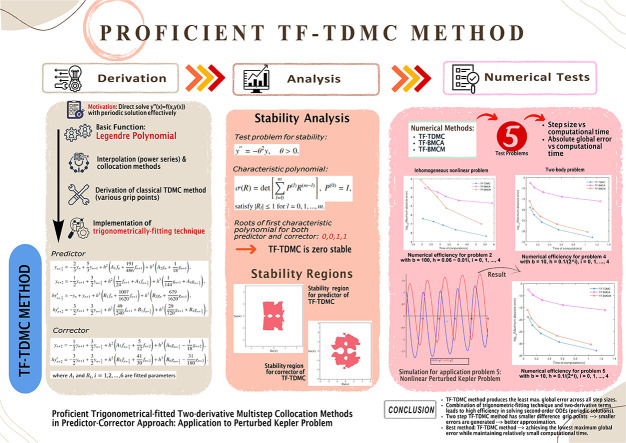

Specifications table

**Table:** 

Subject area:	Mathematics and Statistics
More specific subject area:	*Numerical Analysis*
Name of your method:	*Trigonometrical-fitted two-derivative multistep collocation method in predictor-corrector mode*
Name and reference of original method:	*None*
Resource availability:	*None*

## Background

Second-order ordinary differential equations (ODEs) find extensive utility in forecasting and predicting the evolution of scientific phenomena and application issues, particularly within engineering and physics domains. Examples include their application to astrophysics, control theory, electric circuits, mass-spring system and classical mechanics [[1], [2], [3], [4]]. Numerous studies have been conducted to develop effective techniques for integrating second-order ODEs, particularly those with specific patterns or properties. Fitting techniques are especially popular for handling second-order ODEs with periodic solutions, including trigonometrical fitting, phase and amplification fitting and functional fitting techniques [[5], [6]]. By accurately capturing the oscillatory nature of the problem, these methods ensure that the numerical solution remains close to the exact solution over long time intervals, which is critical for maintaining accuracy in simulations and predictions.

Numerous efficient methods have been developed for solving high-order ODEs and application problems, including the block collocation methods [[7], [8], [9], [10]]. Various collocation methods are developed based on different polynomials and functions in recent years, including Chebyshev polynomials, Block-Pulse functions, Newton-Gregory backward difference polynomial and power series [[11], [12], [13]]. These methods offer the advantage of efficiently handling large differential equations by dividing the problem into smaller, more manageable sub-intervals, which simplifies the numerical solution process and improves accuracy. This approach reduces the computational complexity and error by transforming the differential equations into algebraic equations within each sub-interval, allowing for more precise and stable approximations.

To effectively integrate differential equations with specific solutions like exponential and periodic functions, many researchers have developed linear or block multistep methods that incorporate specialized fitting techniques [[14], [15], [16]]. The fitting techniques in the block collocation method, such as functionally fitted and trigonometric-fitted approaches, enhance accuracy and efficiency by customizing the basis functions to align with the specific characteristics of the differential equations being solved [[17], [18], [19], [20]]. This customization allows for more precise numerical approximations and improved handling of complex problems, such as oscillatory or periodic solutions, leading to better overall performance in solving differential equations. However, there is a lack of advancement in further enhancing existing methods that use fitting techniques. Future improvements could involve integrating multiple enhancements, such as incorporating two-derivative approaches, refining fitting techniques and employing predictor-corrector strategies to boost accuracy and efficiency.

In this study, we propose an explicit fifth-order, two-derivative multistep method using a predictor-corrector approach, denoted as the TF-TDMC method, for the direct integration of second-order ODEs with periodic solutions. The TF-TDMC method is derived using Chebyshev polynomials of up to the fifth order as the basis functions. It involves collocating the differential equation at different points, interpolating the approximate solution through Chebyshev polynomials at the grid points. The method incorporates a trigonometrical-fitting technique to generate frequency-dependent coefficients, enhancing its accuracy for solving second-order ODEs with periodic solutions. Additionally, we provide a comprehensive stability analysis and error norm assessment of the proposed method. The article includes numerical tests comparing the TF-TDMC method with several existing multistep methods, including tests on the notable nonlinear perturbed Kepler problem. The final section concludes with a discussion and summary of the findings.

## Method details

### Derivation of two-derivative multistep collocation method

In this study, we plan to construct efficient fifth-order two-derivative multistep collocation (TDMC) method with predictor-corrector mode to solve special class of second-order ordinary differential equation in the form of

(1) $$y^{\prime \prime }\left(x\right)=f\left(x,y\left(x\right)\right),\;y\left(x_{0}\right)=y_{0},\;y^{\prime }\left(x_{0}\right)={y^{\prime }}_{0}.$$

We begin with the general formulation of TDMC method for solving problem (1), comprises the derivative of the solution $y^{\prime \prime \prime }\left(x\right)=g\left(x,y\left(x\right),y^{\prime }\left(x\right)\right)=g_{x}\left(x,y\left(x\right),y^{\prime }\left(x\right)\right)+g_{y}\left(x,y\left(x\right),y^{\prime }\left(x\right)\right)y^{\prime }+g_{y^{\prime }}\left(x,y\left(x\right),y^{\prime }\left(x\right)\right)f$ as follows:

(2) $$\sum_{j=0}^{k}\alpha_{j}y\left(x+jh\right)+\sum_{j=0}^{m_{1}}\alpha_{v_{j}}y\left(x+v_{j}h\right)=h^{2}\left(\sum_{j=0}^{k}\beta_{j}f_{n+j}+\sum_{j=0}^{m_{2}}\beta_{v_{j}}f_{n+v_{j}}\right)+h^{3}\left(\sum_{j=0}^{k}\gamma_{j}g_{n+j}+\sum_{j=0}^{m_{3}}\gamma_{v_{j}}g_{n+v_{j}}\right),$$

where $\alpha_{j},\alpha_{v_{j}},\beta_{j},\beta_{v_{j}},\gamma_{j},\gamma_{v_{j}}\in R,m_{1},m_{2},m_{3},k\in Z^{+}$ and $v_{j}$ is non-integer.

To construct TDMC method, we approximate the solution by employing the interpolating function with Legendre polynomials serving as basis functions,

(3) $$\begin{array}{ccc} T_{0} & = & 1,\\ T_{1} & = & z,\\ T_{2} & = & \frac{3}{2}z^{2}-\frac{1}{2},\\ T_{3} & = & \frac{5}{2}z^{3}-\frac{3}{2}z,\\ T_{4} & = & \frac{35}{8}z^{4}-\frac{15}{4}z^{2}+\frac{3}{8},\\ T_{5} & = & \frac{63}{8}z^{5}-\frac{35}{4}z^{3}+\frac{15}{8}z. \end{array}$$

Then, we approximate the solution using an interpolating function (power series) in the following equation:

(4) $$Y\left(x\right)=\sum_{j=0}^{s_{1}+s_{2}+\eta -1}a_{j}T_{j}\left(x\right),$$

where $x\in \left[x_{0},b\right],a_{j}$ is unknown parameter, $T_{j}$ is Legendre polynomial, $s_{1}$ and $s_{2}$ are the number of interpolations for second and third derivative respectively, η is distinct collocation point with η>0 and b is endpoints of interval.

Similarly, the second derivative of third derivative of interpolating function will be as follows:

$$Y^{\prime \prime }\left(x\right)=\sum_{j=0}^{s_{1}+s_{2}+\eta -1}a_{j}{T^{\prime \prime }}_{j}\left(x\right),$$

(5) $$Y^{\prime \prime \prime }\left(x\right)=\sum_{j=0}^{s_{1}+s_{2}+\eta -1}a_{j}{T^{\prime \prime \prime }}_{j}\left(x\right).$$

The continuous approximation is formulated by enforcing the following conditions:

(6) $$\begin{array}{ccc} Y(x_{n+\eta }) & = & y_{n+\eta },\;\;\eta =0,1,\frac{5}{3},\\ Y^{\prime \prime }\left(x_{n+s_{1}}\right) & = & f_{n+s_{1}},\;\;\;s_{1}=0,1,\frac{5}{3},2,\\ Y^{\prime \prime \prime }\left(x_{n+s_{2}}\right) & = & g_{n+s_{2}},\;\;\;s_{2}=0,1,\frac{5}{3},2. \end{array}$$

At first, we develop predictor formulae for TDMC method, we solve three set of equations based on (4) and (5) to obtain the predictor based on the grid points, $\frac{5}{3}$ and 2. We solve $Y\left(x_{n}\right),Y\left(x_{n+1}\right),Y^{\prime \prime }\left(x_{n}\right),Y^{\prime \prime }\left(x_{n+1}\right),Y^{\prime \prime \prime }\left(x_{n}\right),Y^{\prime \prime \prime }\left(x_{n+1}\right)$ simultaneously to obtain coefficients $a_{j},\;j=0,1,...,5,$ then substituting the values $a_{j}$ into Eq. (4) and yield the continuous method as follows:

(7) $$Y\left(x\right)=\sum_{0}^{1}\alpha_{j}y_{n+j}+h^{2}\sum_{0}^{1}\beta_{j}f_{n+j}+h^{3}\sum_{0}^{1}\gamma_{j}g_{n+j}.$$

Later, we express $\alpha_{j},\;\beta_{j},\;\gamma_{j}$ as continuous coefficients in terms of t by letting $t=\frac{x-x_{n}}{h}$, the following parameters as obtained as

(8) $$\begin{array}{ccc} & & \alpha_{0}=1-t,\;\alpha_{1}=t,\\ & & \beta_{0}=-\frac{7}{20}t+\frac{1}{2}t^{2}-\frac{1}{4}t^{4}+\frac{1}{10}t^{5},\\ & & \beta_{1}=-\frac{3}{20}t+\frac{1}{4}t^{4}-\frac{1}{10}t^{5},\\ & & \gamma_{0}=-\frac{1}{20}t+\frac{1}{6}t^{3}-\frac{1}{6}t^{4}+\frac{1}{20}t^{5},\\ & & \gamma_{1}=\frac{1}{30}t-\frac{1}{12}t^{4}+\frac{1}{20}t^{5}. \end{array}$$

Let $t=\frac{5}{3},$ we get

(9) $$y_{n+\frac{5}{3}}=-\frac{2}{3}y_{n}+\frac{5}{3}y_{n+1}+h^{2}\left(\frac{79}{486}f_{n}+\frac{191}{486}f_{n+1}\right)+h^{3}\left(\frac{11}{243}g_{n}+\frac{1}{18}g_{n+1}\right).$$

To obtain the equation for ${y^{\prime }}_{n+\frac{5}{3}},$ Eq. (7) is differentiated with respect to x, substituted by $t=\frac{x-x_{n}}{h}$ and setting $t=\frac{5}{3},$ resulting in:

(10) $${y^{\prime }}_{n+\frac{5}{3}}=\frac{1}{h}\left[-y_{n}+y_{n+1}+h^{2}\left(\frac{883}{1620}f_{n}+\frac{1007}{1620}f_{n+1}\right)+h^{3}\left(\frac{49}{270}g_{n}+\frac{679}{1620}g_{n+1}\right)\right].$$

Similarly, we simultaneously solve for $Y\left(x_{n+1}\right),\;Y\left(x_{n+5/3}\right),Y^{\prime \prime }\left(x_{n+1}\right),Y^{\prime \prime }\left(x_{n+5/3}\right),Y^{\prime \prime \prime }\left(x_{n+1}\right),\;Y^{\prime \prime \prime }\left(x_{n+5/3}\right)$ to derive new coefficients $a_{j},\;j=0,1,...,5$. Subsequently, by substituting these $a_{j}$ into Eq. (4), we derived continuous method successfully as follows:

(11) $$Y\left(x\right)=\alpha_{1}y_{n+1}+\alpha_{5/3}y_{n+5/3}+h^{2}\left(\beta_{1}f_{n+1}+\beta_{5/3}f_{n+5/3}\right)+h^{3}\left(\gamma_{1}g_{n+1}+\gamma_{5/3}g_{n+5/3}\right).$$

Subsequently, when we define $\alpha_{k},\;\beta_{k},\;\gamma_{k},\;\;k=1,\frac{5}{3}$, as continuous functions of t by setting $t=\frac{x-x_{n}}{h}$, we acquire

(12) $$\begin{array}{ccc} & & \alpha_{1}=\frac{5}{2}-\frac{3}{2}t,\;\alpha_{5/3}=-\frac{3}{2}+\frac{3}{2}t,\\ & & \beta_{1}=-\frac{1}{6}+\frac{649}{240}t-\frac{25}{4}t^{2}+\frac{45}{8}t^{3}-\frac{9}{4}t^{4}+\frac{27}{80}t^{5},\\ & & \beta_{5/3}=1-\frac{323}{80}t+\frac{27}{4}t^{2}-\frac{45}{8}t^{3}+\frac{9}{4}t^{4}-\frac{27}{80}t^{5},\\ & & \gamma_{1}=-\frac{73}{144}+\frac{1469}{720}t-\frac{25}{8}t^{2}+\frac{55}{24}-\frac{13}{16}t^{4}+\frac{9}{80}t^{5},\\ & & \gamma_{5/3}=-\frac{109}{432}+\frac{2327}{2160}t-\frac{15}{8}t^{2}+\frac{13}{8}-\frac{11}{16}t^{4}+\frac{9}{80}t^{5}. \end{array}$$

When t=2, we obtain $y_{n+2}$ and its derivative (differentiate Eq. (11) with respect to x and $t=\frac{x-x_{n}}{h}$) as follows:

(13) $$\begin{array}{ccc} & & y_{n+2}=-\frac{1}{2}y_{n+1}+\frac{3}{2}y_{n+5/3}+h^{2}\left(\frac{1}{24}f_{n+1}+\frac{1}{8}f_{n+5/3}\right)+h^{3}\left(\frac{1}{144}g_{n+1}+\frac{1}{432}g_{n+5/3}\right),\\ & & {y^{\prime }}_{n+2}=\frac{1}{h}\left[-\frac{3}{2}y_{n+1}+\frac{3}{2}y_{n+5/3}+h^{2}\left(\frac{49}{240}f_{n+1}+\frac{37}{80}f_{n+5/3}\right)+h^{3}\left(\frac{29}{720}g_{n+1}+\frac{167}{2160}g_{n+5/3}\right)\right]. \end{array}$$

To derive the equation for the corrector, we simultaneously solve for $Y\left(x_{n+1}\right),\;Y\left(x_{n+5/3}\right),Y^{\prime \prime }\left(x_{n+5/3}\right),Y^{\prime \prime }\left(x_{n+2}\right),$

$Y^{\prime \prime \prime }\left(x_{n+5/3}\right),\;Y^{\prime \prime \prime }\left(x_{n+2}\right)$. Using the similar ways, we obtain

(14) $$\begin{array}{ccc} & & y_{n+2}=-\frac{1}{2}y_{n+1}+\frac{3}{2}y_{n+5/3}+h^{2}\left(-\frac{1}{4}f_{n+5/3}+\frac{5}{12}f_{n+2}\right)+h^{3}\left(-\frac{11}{108}g_{n+5/3}-\frac{1}{18}g_{n+2}\right),\\ & & {y^{\prime }}_{n+2}=\frac{1}{h}\left[-\frac{3}{2}y_{n+1}+\frac{3}{2}y_{n+5/3}+h^{2}\left(-\frac{7}{10}f_{n+5/3}+\frac{41}{30}f_{n+2}\right)+h^{3}\left(-\frac{163}{540}g_{n+5/3}-\frac{31}{180}g_{n+2}\right)\right]. \end{array}$$

Therefore, the complete formula for the corrector is provided below:

(15) $$\begin{array}{ccc} & & y_{n+\frac{5}{3}}=-\frac{2}{3}y_{n}+\frac{5}{3}y_{n+1}+h^{2}\left(\frac{79}{486}f_{n}+\frac{191}{486}f_{n+1}\right)+h^{3}\left(\frac{11}{243}g_{n}+\frac{1}{18}g_{n+1}\right),\\ & & h{y^{\prime }}_{n+\frac{5}{3}}=-y_{n}+y_{n+1}+h^{2}\left(\frac{883}{1620}f_{n}+\frac{1007}{1620}f_{n+1}\right)+h^{3}\left(\frac{49}{270}g_{n}+\frac{679}{1620}g_{n+1}\right),\\ & & y_{n+2}=-\frac{1}{2}y_{n+1}+\frac{3}{2}y_{n+\frac{5}{3}}+h^{2}\left(\frac{1}{24}f_{n+1}+\frac{1}{8}f_{n+\frac{5}{3}}\right)+h^{3}\left(\frac{1}{144}g_{n+1}+\frac{1}{432}g_{n+\frac{5}{3}}\right),\\ & & h{y^{\prime }}_{n+2}=-\frac{3}{2}y_{n+1}+\frac{3}{2}y_{n+\frac{5}{3}}+h^{2}\left(\frac{49}{240}f_{n+1}+\frac{37}{80}f_{n+\frac{5}{3}}\right)+h^{3}\left(\frac{29}{720}g_{n+1}+\frac{167}{2160}g_{n+\frac{5}{3}}\right), \end{array}$$

Corrector formula:

(16) $$\begin{array}{ccc} & & y_{n+2}=-\frac{1}{2}y_{n+1}+\frac{3}{2}y_{n+5/3}+h^{2}\left(-\frac{1}{4}f_{n+5/3}+\frac{5}{12}f_{n+2}\right)+h^{3}\left(-\frac{11}{108}g_{n+5/3}-\frac{1}{18}g_{n+2}\right),\\ & & h{y^{\prime }}_{n+2}=-\frac{3}{2}y_{n+1}+\frac{3}{2}y_{n+5/3}+h^{2}\left(-\frac{7}{10}f_{n+5/3}+\frac{41}{30}f_{n+2}\right)+h^{3}\left(-\frac{163}{540}g_{n+5/3}-\frac{31}{180}g_{n+2}\right). \end{array}$$

### Trigonometrically-Fitted two-derivative linear multistep method

In developing the TDMC method with a trigonometric fitting technique, denoted as TF-TDMC method, we replace some coefficients in TDMC method with $A_{i}$ and $B_{i},\;i=1,2,...,6$ as follows:

(17) $$\begin{array}{ccc} & & y_{n+\frac{5}{3}}=-\frac{2}{3}y_{n}+\frac{5}{3}y_{n+1}+h^{2}\left(A_{1}f_{n}+\frac{191}{486}f_{n+1}\right)+h^{3}\left(A_{2}g_{n}+\frac{1}{18}g_{n+1}\right),\\ & & y_{n+2}=-\frac{1}{2}y_{n+1}+\frac{3}{2}y_{n+\frac{5}{3}}+h^{2}\left(\frac{1}{24}f_{n+1}+A_{3}f_{n+\frac{5}{3}}\right)+h^{3}\left(\frac{1}{144}g_{n+1}+A_{4}g_{n+\frac{5}{3}}\right),\\ & & h{y^{\prime }}_{n+\frac{5}{3}}=-y_{n}+y_{n+1}+h^{2}\left(B_{1}f_{n}+\frac{1007}{1620}f_{n+1}\right)+h^{3}\left(B_{2}g_{n}+\frac{679}{1620}g_{n+1}\right),\\ & & h{y^{\prime }}_{n+2}=-\frac{3}{2}y_{n+1}+\frac{3}{2}y_{n+\frac{5}{3}}+h^{2}\left(\frac{49}{240}f_{n+1}+B_{3}f_{n+\frac{5}{3}}\right)+h^{3}\left(\frac{29}{720}g_{n+1}+B_{4}g_{n+\frac{5}{3}}\right), \end{array}$$

(18) $$\begin{array}{ccc} & & y_{n+2}=-\frac{1}{2}y_{n+1}+\frac{3}{2}y_{n+5/3}+h^{2}\left(A_{5}f_{n+5/3}+\frac{5}{12}f_{n+2}\right)+h^{3}\left(A_{6}g_{n+5/3}-\frac{1}{18}g_{n+2}\right),\\ & & h{y^{\prime }}_{n+2}=-\frac{3}{2}y_{n+1}+\frac{3}{2}y_{n+5/3}+h^{2}\left(B_{5}f_{n+5/3}+\frac{41}{30}f_{n+2}\right)+h^{3}\left(B_{6}g_{n+5/3}-\frac{31}{180}g_{n+2}\right). \end{array}$$

We set $y_{n+k}=e^{i\theta x}e^{kiw},\;k=0,1,\frac{5}{3},2$ where i is imaginary unit,w=θh and θ∈R. Subsequently, we determine the first derivative, ${y^{\prime }}_{n+k}$, second derivative, $f_{n+k}$ and third derivative, $g_{n+k}$ using the formula of $y_{n+k}$ as mentioned above. Then, exponential functions $e^{i\theta x}$ and $e^{-i\theta x}$ are integrated at each stage, we obtain the equations corresponding to $y,\;hy^{\prime }$ below:

(19) $$\begin{array}{ccc} & & e^{\pm \frac{5}{3}iw}=-\frac{2}{3}+\frac{5}{3}e^{\pm iw}-w^{2}\left(A_{1}+\frac{191}{486}e^{\pm iw}\right)\mp iw^{3}\left(A_{2}+\frac{1}{18}e^{\pm iw}\right),\\ & & e^{\pm 2iw}=-\frac{1}{2}e^{\pm iw}+\frac{3}{2}e^{\pm \frac{5}{3}iw}-w^{2}\left(\frac{1}{24}e^{\pm iw}+A_{3}e^{\pm \frac{5}{3}iw}\right)\mp iw^{3}\left(\frac{1}{144}e^{\pm iw}+A_{4}e^{\pm \frac{5}{3}iw}\right),\\ & & e^{\pm 2iw}=-\frac{1}{2}e^{\pm iw}+\frac{3}{2}e^{\pm \frac{5}{3}iw}-w^{2}\left(A_{5}e^{\pm \frac{5}{3}iw}+\frac{5}{12}e^{\pm 2iw}\right)\mp iw^{3}\left(A_{6}e^{\pm \frac{5}{3}iw}-\frac{1}{18}e^{\pm 2iw}\right),\\ & & \pm iwe^{\pm \frac{5}{3}iw}=-1+e^{\pm iw}-w^{2}\left(B_{1}+\frac{1007}{1620}e^{\pm iw}\right)\mp iw^{3}\left(B_{2}+\frac{679}{1620}e^{\pm iw}\right),\\ & & \pm iwe^{\pm 2iw}=-\frac{3}{2}e^{\pm iw}+\frac{3}{2}e^{\pm \frac{5}{3}iw}-w^{2}\left(\frac{49}{240}e^{\pm iw}+B_{3}e^{\pm \frac{5}{3}iw}\right)\mp iw^{3}\left(\frac{29}{720}e^{\pm iw}+B_{4}e^{\pm \frac{5}{3}iw}\right),\\ & & \pm iwe^{\pm 2iw}=-\frac{3}{2}e^{\pm iw}+\frac{3}{2}e^{\pm \frac{5}{3}iw}-w^{2}\left(B_{3}e^{\pm \frac{5}{3}iw}+\frac{41}{30}e^{\pm 2iw}\right)\mp iw^{3}\left(B_{4}e^{\pm \frac{5}{3}iw}-\frac{31}{180}e^{\pm 2iw}\right). \end{array}$$

The relation $\cos\left(w\right)=\frac{e^{iw}+e^{-iw}}{2}$ and $\sin\left(w\right)=\frac{e^{iw}-e^{-iw}}{2i}$ are substituted in the equations Eq. (19) corresponding to y, we get trigonometric functions of w below:

(20) $$\begin{array}{ccc} & & \cos\left(\frac{5}{3}w\right)=-\frac{2}{3}+\frac{5}{3}\cos\left(w\right)-w^{2}\left[A_{1}+\frac{191}{486}\cos\left(w\right)\right]+\frac{1}{18}w^{3}\sin\left(w\right),\\ & & \sin\left(\frac{5}{3}w\right)=\frac{5}{3}\sin\left(w\right)-\frac{191}{486}w^{2}\sin\left(w\right)-w^{3}\left[A_{2}+\frac{1}{18}\cos\left(w\right)\right],\\ & & \cos\left(2w\right)=-\frac{1}{2}\cos\left(w\right)+\frac{3}{2}\cos\left(\frac{5}{3}w\right)-w^{2}\left[\frac{1}{24}\cos\left(w\right)+A_{3}\cos\left(\frac{5}{3}w\right)\right]+w^{3}\left[\frac{1}{144}\sin\left(w\right)+A_{4}\sin\left(\frac{5}{3}w\right)\right],\\ & & \sin\left(2w\right)=-\frac{1}{2}\sin\left(w\right)+\frac{3}{2}\sin\left(\frac{5}{3}w\right)-w^{2}\left[\frac{1}{24}\sin\left(w\right)+A_{3}\sin\left(\frac{5}{3}w\right)\right]-w^{3}\left[\frac{1}{144}\cos\left(w\right)+A_{4}\cos\left(\frac{5}{3}w\right)\right],\\ & & \cos\left(2w\right)=-\frac{1}{2}\cos\left(w\right)+\frac{3}{2}\cos\left(\frac{5}{3}w\right)-w^{2}\left[A_{5}\cos\left(\frac{5}{3}w\right)+\frac{5}{12}\cos\left(2w\right)\right]+w^{3}\left[A_{6}\sin\left(\frac{5}{3}w\right)-\frac{1}{18}\sin\left(2w\right)\right],\\ & & \sin\left(2w\right)=-\frac{1}{2}\sin\left(w\right)+\frac{3}{2}\sin\left(\frac{5}{3}w\right)-w^{2}\left[A_{5}\sin\left(\frac{5}{3}w\right)+\frac{5}{12}\sin\left(2w\right)\right]-w^{3}\left[A_{6}\cos\left(\frac{5}{3}w\right)-\frac{1}{18}\cos\left(2w\right)\right], \end{array}$$

Further solving Eq. (20) and apply Taylor series expansion, we obtain the frequency-dependent parameters of $A_{i},\;i=1,2,...,6.$

(21) $$\begin{array}{ccc} & & A_{1}=\frac{79}{486}+\frac{191}{104976}w^{4}-\frac{56411}{132269760}w^{6}+\frac{1043333}{42855402240}w^{8}-\frac{44178307}{63640272326400}w^{10}+O\left(w^{12}\right),\\ & & A_{2}=\frac{11}{243}+\frac{919}{787320}w^{4}-\frac{10153}{89282088}w^{6}+\frac{56603}{12856620672}w^{8}-\frac{4655327}{47730204244800}w^{10}+O\left(w^{12}\right),\\ & & A_{3}=\frac{1}{8}-\frac{11}{174960}w^{4}-\frac{89}{29393280}w^{6}+\frac{113}{2645395200}w^{8}+\frac{26027}{84853696435200}w^{10}+O\left(w^{12}\right),\\ & & A_{4}=\frac{1}{432}-\frac{1}{58320}w^{4}+\frac{949}{2380855680}w^{6}-\frac{277}{71425670400}w^{8}+\frac{29}{1346884070400}w^{10}+O\left(w^{12}\right),\\ & & A_{5}=-\frac{1}{4}+\frac{67}{349920}w^{4}-\frac{47}{29393280}w^{6}+\frac{31}{5290790400}w^{8}-\frac{589}{42426848217600}w^{10}+O\left(w^{12}\right),\\ & & A_{6}=-\frac{11}{108}+\frac{1}{116640}w^{4}-\frac{11}{340122240}w^{6}+\frac{1}{142851340800}w^{8}+\frac{1}{4714094246400}w^{10}+O\left(w^{12}\right). \end{array}$$

In a similar manner, we incorporate the relationship between cos(w) and sin(w) into the equations Eq. (19), which correspond to $hy^{\prime }$. As a result, we obtain trigonometric functions of w as follows:

(22) $$\begin{array}{ccc} & & w\sin\left(\frac{5}{3}w\right)=1-\cos\left(w\right)+w^{2}\left[B_{1}+\frac{1007}{1620}\cos\left(w\right)\right]-\frac{679}{1620}w^{3}\sin\left(w\right),\\ & & w\cos\left(\frac{5}{3}w\right)=\sin\left(w\right)-\frac{1007}{1620}w^{2}\sin\left(w\right)-w^{3}\left[B_{2}+\frac{679}{1620}\cos\left(w\right)\right],\\ & & w\sin\left(2w\right)=\frac{3}{2}\cos\left(w\right)-\frac{3}{2}\cos\left(\frac{5}{3}w\right)+w^{2}\left[\frac{49}{240}\cos\left(w\right)+B_{3}\cos\left(\frac{5}{3}w\right)\right]-w^{3}\left[\frac{29}{720}\sin\left(w\right)+B_{4}\sin\left(\frac{5}{3}w\right)\right],\\ & & w\cos\left(2w\right)=-\frac{3}{2}\sin\left(w\right)+\frac{3}{2}\sin\left(\frac{5}{3}w\right)-w^{2}\left[\frac{49}{240}\sin\left(w\right)+B_{3}\sin\left(\frac{5}{3}w\right)\right]-w^{3}\left[\frac{29}{720}\cos\left(w\right)+B_{4}\cos\left(\frac{5}{3}w\right)\right],\\ & & w\sin\left(2w\right)=\frac{3}{2}\cos\left(w\right)-\frac{3}{2}\cos\left(\frac{5}{3}w\right)+w^{2}\left[B_{5}\cos\left(\frac{5}{3}w\right)+\frac{41}{30}\cos\left(2w\right)\right]-w^{3}\left[B_{6}\sin\left(\frac{5}{3}w\right)-\frac{31}{180}\sin\left(2w\right)\right],\\ & & w\cos\left(2w\right)=\frac{3}{2}\sin\left(w\right)+\frac{3}{2}\sin\left(\frac{5}{3}w\right)-w^{2}\left[B_{5}\sin\left(\frac{5}{3}w\right)+\frac{41}{30}\sin\left(2w\right)\right]-w^{3}\left[B_{6}\cos\left(\frac{5}{3}w\right)-\frac{31}{180}\cos\left(2w\right)\right]. \end{array}$$

Subsequently, the coefficients above are used to generate parameters$B_{i},\;i=1,2,...,6$ through Taylor series expansion.

(23) $$\begin{array}{ccc} & & B_{1}=\frac{883}{1620}+\frac{1169}{116640}w^{4}-\frac{1193447}{1193447}w^{6}+\frac{24941041}{142851340800}w^{8}-\frac{394347413}{70711413696000}w^{10}+O\left(w^{12}\right),\\ & & B_{2}=\frac{49}{270}+\frac{127241}{18370800}w^{4}-\frac{25409}{33067440}w^{6}+\frac{15764641}{471409424640}w^{8}-\frac{1712206627}{2068308850608000}w^{10}+O\left(w^{12}\right),\\ & & B_{3}=\frac{37}{80}+\frac{23}{43740}w^{4}-\frac{383}{18370800}w^{6}+\frac{367}{1322697600}w^{8}-\frac{205327}{106067120544000}w^{10}+O\left(w^{12}\right),\\ & & B_{4}=\frac{167}{2160}-\frac{251}{2041200}w^{4}+\frac{1259}{476171136}w^{6}-\frac{19463}{785682374400}w^{8}+\frac{82477}{612832252032000}w^{10}+O\left(w^{12}\right),\\ & & B_{5}=-\frac{7}{10}+\frac{101}{174960}w^{4}-\frac{89}{18370800}w^{6}+\frac{47}{2645395200}w^{8}-\frac{557}{13258390068000}w^{10}+O\left(w^{12}\right),\\ & & B_{6}=-\frac{163}{540}+\frac{107}{4082400}w^{4}-\frac{239}{2380855680}w^{6}+\frac{47}{1571364748800}w^{8}+\frac{191}{306416126016000}w^{10}+O\left(w^{12}\right). \end{array}$$

As w approaches 0, the coefficients$A_{i}$ and $B_{i},\;i=1,2,...,6$ of the proposed methods using the fitting technique will return to their classical form values.

## Method validation

### Stability analysis of TF-TDMC method

Here, we discuss the zero stability of block collocation method for solving second-order ODEs defined by [8] as follows:

Definition 1 (Zero stable) A block multistep method with order p is zero stable provided the roots, $R_{l}$ for l=0,1,...,m of the characteristic polynomial, σ(R) such that:

(24) $$\sigma \left(R\right)=\det\left[\sum_{l=0}^{m}P^{\left(l\right)}R^{\left(m-l\right)}\right],P^{\left(0\right)}=I,$$

satisfy $|R_{l}|\leq 1$ for l=0,1,...,m. If $R_{l}$ is a repeated root, then the multiplicity of the root of modulus 1 must be at most 2, where I is identity matrix and $P^{\left(l\right)}$ is m×m matrix.

First, we use second-order test problem as follow:

(25) $$y^{\prime \prime }=-\theta^{2}y,\;\;\theta >0.$$

Apply predictor formulae of TF-TDMC method into the test problem above and substitute w=θh, we obtain

(26) $$y_{n+\frac{5}{3}}=\left(-\frac{2}{3}-A_{1}w^{2}\right)y_{n}+\left(\frac{5}{3}-\frac{191}{486}w^{2}\right)y_{n+1}+\left(-A_{2}w^{2}\right)h{y^{\prime }}_{n}+\left(-\frac{1}{18}w^{2}\right)h{y^{\prime }}_{n+1},$$

(27) $$h{y^{\prime }}_{n+\frac{5}{3}}=\left(-1-B_{1}w^{2}\right)y_{n}+\left(1-\frac{1007}{1620}w^{2}\right)y_{n+1}+\left(-B_{2}w^{2}\right)h{y^{\prime }}_{n}+\left(-\frac{679}{1620}w^{2}\right)h{y^{\prime }}_{n+1},$$

(28) $$y_{n+2}=\left(-\frac{1}{2}-\frac{1}{24}w^{2}\right)y_{n+1}+\left(\frac{3}{2}-A_{3}w^{2}\right)y_{n+\frac{5}{3}}+\left(-\frac{1}{144}w^{2}\right)h{y^{\prime }}_{n+1}+\left(-A_{4}w^{2}\right)h{y^{\prime }}_{n+\frac{5}{3}},$$

(29) $$h{y^{\prime }}_{n+2}=\left(-\frac{3}{2}-\frac{49}{240}w^{2}\right)y_{n+1}+\left(\frac{3}{2}-B_{3}w^{2}\right)y_{n+\frac{5}{3}}+\left(-\frac{1}{144}w^{2}\right)h{y^{\prime }}_{n+1}+\left(-B_{4}w^{2}\right)h{y^{\prime }}_{n+\frac{5}{3}}.$$

We transform Eqs. (26)–(29) into the matrix form as below:

(30) $$\left(\begin{array}{cc} 1 & 0\\ 0 & 1 \end{array}\right)\left(\begin{array}{c} y_{n+2}\\ h{y^{\prime }}_{n+2} \end{array}\right)=\left(\begin{array}{cc} M_{11} & M_{12}\\ M_{21} & M_{22} \end{array}\right)\left(\begin{array}{c} y_{n+1}\\ h{y^{\prime }}_{n+1} \end{array}\right)+\left(\begin{array}{cc} N_{11} & N_{12}\\ N_{21} & N_{22} \end{array}\right)\left(\begin{array}{c} y_{n}\\ h{y^{\prime }}_{n} \end{array}\right),$$

where

(31) $$\begin{array}{ccc} & & M_{11}=2-\frac{1091}{1296}w^{2}+\frac{35387}{699840}w^{4}-\frac{23}{262440}w^{6}+\frac{229}{12247200}w^{8}-\frac{3893719}{3856986201600}w^{10}+O\left(w^{12}\right),\\ & & M_{12}=-\frac{13}{144}w^{2}+\frac{3211}{349920}w^{4}+\frac{7679}{755827200}w^{8}-\frac{2666717}{440798423040}w^{10}+O\left(w^{12}\right),\\ & & M_{21}=1-\frac{10639}{6480}w^{2}+\frac{804199}{699840}w^{4}-\frac{13841}{18370800}w^{6}+\frac{119279}{734832000}w^{8}-\frac{42351737}{6060978316800}w^{10}+O\left(w^{12}\right),\\ & & M_{22}=-\frac{13}{144}w^{2}+\frac{87691}{874800}w^{4}+\frac{18134719}{26453952000}w^{8}-\frac{16036448009}{77139724032000}w^{10}+O\left(w^{12}\right),\\ & & N_{11}=-1-\frac{205}{1296}w^{2}+\frac{15103}{699840}w^{4}-\frac{2839}{1049760}w^{6}+\frac{522973}{587865600}w^{8}-\frac{1486403507}{15427944806400}w^{10}+O\left(w^{12}\right),\\ & & N_{12}=-\frac{11}{162}w^{2}+\frac{11}{1944}w^{4}-\frac{919}{524880}w^{6}+\frac{126713}{396809280}w^{8}-\frac{898069}{42855402240}w^{10}+O\left(w^{12}\right),\\ & & N_{21}=-1+\frac{919}{6480}w^{2}+\frac{410531}{3499200}w^{4}-\frac{91913}{36741600}w^{6}+\frac{316789}{139968000}w^{8}-\frac{377458272749}{848536964352000}w^{10}+O\left(w^{12}\right),\\ & & N_{22}=-\frac{11}{162}w^{2}+\frac{407}{19440}w^{4}-\frac{919}{524880}w^{6}+\frac{2913509}{3968092800}w^{8}-\frac{12887129}{214277011200}w^{10}+O\left(w^{12}\right). \end{array}$$

We substitute $\left(\begin{array}{c} y_{n+2}\\ h{y^{\prime }}_{n+2} \end{array}\right)=R^{2},\;\left(\begin{array}{c} y_{n+1}\\ h{y^{\prime }}_{n+1} \end{array}\right)=R$ and $\left(\begin{array}{c} y_{n}\\ h{y^{\prime }}_{n} \end{array}\right)=1$, we yield the following first characteristic polynomial

(32) $$\;\sigma \left(R,w\right)=\left(\begin{array}{cc} 1 & 0\\ 0 & 1 \end{array}\right)R^{2}-\left(\begin{array}{cc} M_{11} & M_{12}\\ M_{21} & M_{22} \end{array}\right)R-\left(\begin{array}{cc} N_{11} & N_{12}\\ N_{21} & N_{22} \end{array}\right).$$

Then we determine the determinant of the first characteristic polynomial and set w=0. This results in the following stability polynomial:

(33) $$R^{4}-2R^{3}+R^{2}=0.$$

Hence, the roots of stability polynomial are 0,0,1,1. All of the roots have modulus less or equal to one, which satisfied the zero stable conditions given in Definition 1. Thus, we conclude that the predictor formulae of TF-TDMC method is zero stable.

**Fig. 1:**
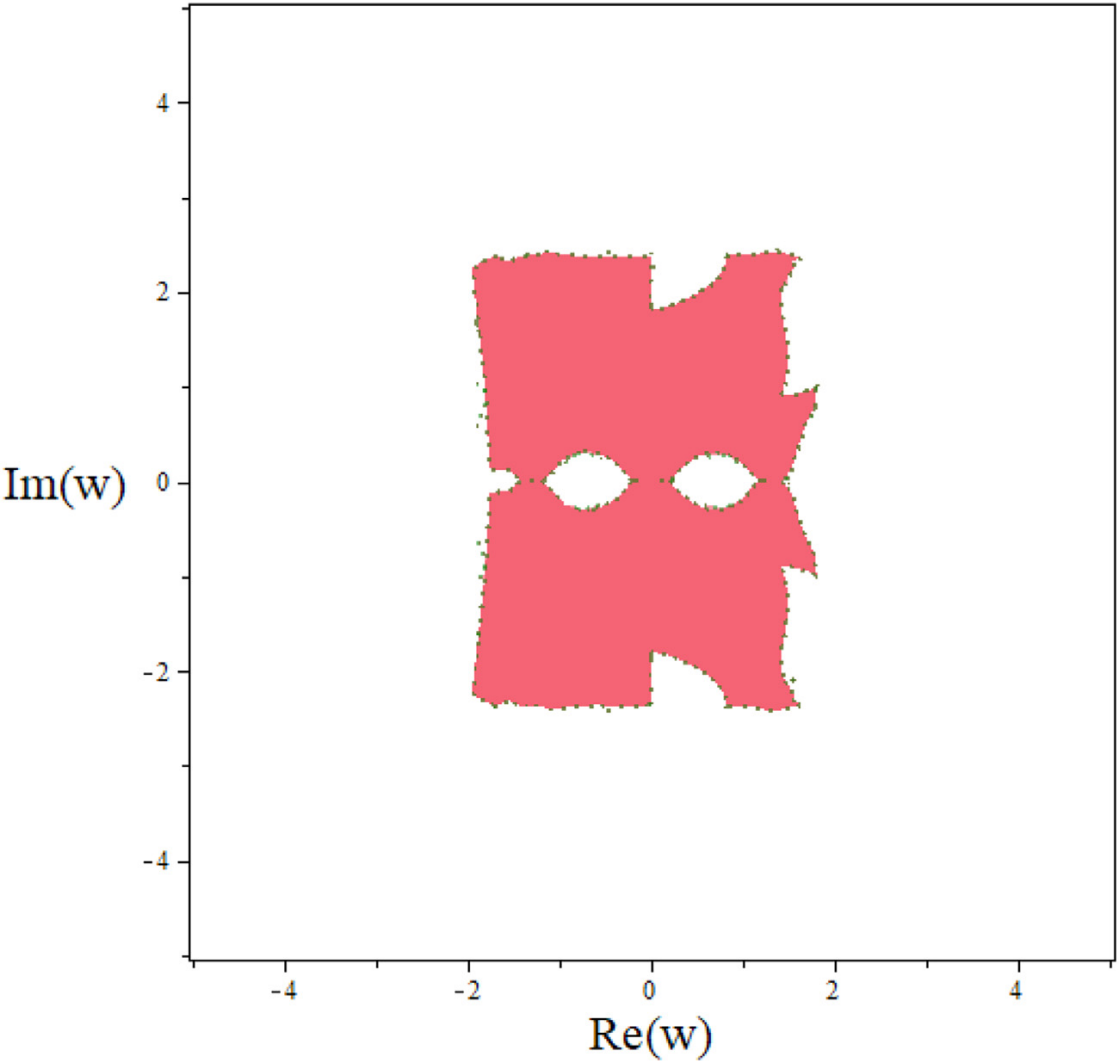
The stability region of predictor for TF-TDMC method.

Next, we let σ(R,w)=0 and solve for R in term of w within the matrice, resulting in 2×2 matrix P(w). The stability region in complex plane of TF-TDMC method can be defined as

(34) $$R_{S}=\left\{w:\left|\lambda_{i}\left(P\left(w\right)\right)\right|<1,\;i=1,2\right\},$$

where $\lambda_{i}$ are eigenvalues of P(w). The stability region of predictor for TF-TDMC method is shown in Fig. 1.

Similarly, for analyzing corrector of TF-TDMC method, we can simply transform into the matrix form:

(35) $$\left(1+\frac{5}{12}w^{2}\right)y_{n+2}=\left(-\frac{1}{2}\right)y_{n+1}+\left(\frac{3}{2}-A_{5}w^{2}\right)y_{n+\frac{5}{3}}+\left(-A_{6}w^{2}\right)h{y^{\prime }}_{n+\frac{5}{3}},$$

(36) $$\left(1-\frac{31}{180}w^{2}\right)h{y^{\prime }}_{n+2}=\left(-\frac{3}{2}\right)y_{n+1}+\left(\frac{3}{2}-B_{5}w^{2}\right)y_{n+\frac{5}{3}}+\left(-\frac{41}{30}w^{2}\right)y_{n+2}+\left(-B_{6}w^{2}\right)h{y^{\prime }}_{n+\frac{5}{3}}.$$

Substitute (26), (27), (28), (29) into (35), (36) and do some arrangement, we yield

(37) $$\left(\begin{array}{cc} 1 & 0\\ 0 & 1 \end{array}\right)\left(\begin{array}{c} y_{n+2}\\ h{y^{\prime }}_{n+2} \end{array}\right)=\left(\begin{array}{cc} {\hat{M}}_{11} & {\hat{M}}_{12}\\ {\hat{M}}_{21} & {\hat{M}}_{22} \end{array}\right)\left(\begin{array}{c} y_{n+1}\\ h{y^{\prime }}_{n+1} \end{array}\right)+\left(\begin{array}{cc} {\hat{N}}_{11} & {\hat{N}}_{12}\\ {\hat{N}}_{21} & {\hat{N}}_{22} \end{array}\right)\left(\begin{array}{c} y_{n}\\ h{y^{\prime }}_{n} \end{array}\right),$$

where

(38) $$\begin{array}{ccc} & & {\hat{M}}_{11}=-2-\frac{275}{324}w^{2}+\frac{35299}{349920}w^{4}-\frac{931933}{31492800}w^{6}+\frac{436363}{35271936}w^{8}-\frac{198495464521}{38569862016000}w^{10}+O\left(w^{12}\right),\\ & & {\hat{M}}_{12}=-\frac{1}{12}w^{2}-\frac{28829}{349920}w^{4}+\frac{2512939}{62985600}w^{6}-\frac{4440649}{251942400}w^{8}+\frac{590711588113}{77139724032000}w^{10}+O\left(w^{12}\right),\\ & & {\hat{M}}_{21}=1-\frac{545}{324}w^{2}+\frac{696443}{1749600}w^{4}-\frac{847921}{551124000}w^{6}+\frac{10529609}{99202320000}w^{8}-\frac{49162122487}{5303356027200000}w^{10}+O\left(w^{12}\right),\\ & & {\hat{M}}_{22}=-\frac{1}{12}w^{2}-\frac{386497}{1749600}w^{4}-\frac{15930937}{314928000}w^{6}-\frac{4634705389}{396809280000}w^{8}-\frac{2331023100443}{1928493100800000}w^{10}+O\left(w^{12}\right),\\ & & {\hat{N}}_{11}=-1-\frac{49}{324}w^{2}-\frac{8839}{349920}w^{4}+\frac{113767}{7873200}w^{6}-\frac{30691}{4408992}w^{8}+\frac{130045338841}{38569862016000}w^{10}+O\left(w^{12}\right),\\ & & {\hat{N}}_{12}=-\frac{11}{162}w^{2}+\frac{77}{5832}w^{4}-\frac{799}{131220}w^{6}+\frac{461737}{198404640}w^{8}-\frac{48594977}{53569252800}w^{10}+O\left(w^{12}\right),\\ & & {\hat{N}}_{21}=-1+\frac{59}{324}w^{2}-\frac{53843}{1749600}w^{4}-\frac{40906333}{1102248000}w^{6}-\frac{1239982543}{198404640000}w^{8}-\frac{6437720654063}{5303356027200000}w^{10}+O\left(w^{12}\right),\\ & & {\hat{N}}_{22}=-\frac{11}{162}w^{2}+\frac{1441}{29160}w^{4}-\frac{1703}{1749600}w^{6}+\frac{31841221}{19840464000}w^{8}-\frac{934727497}{10713850560000}w^{10}+O\left(w^{12}\right). \end{array}$$

**Fig. 2:**
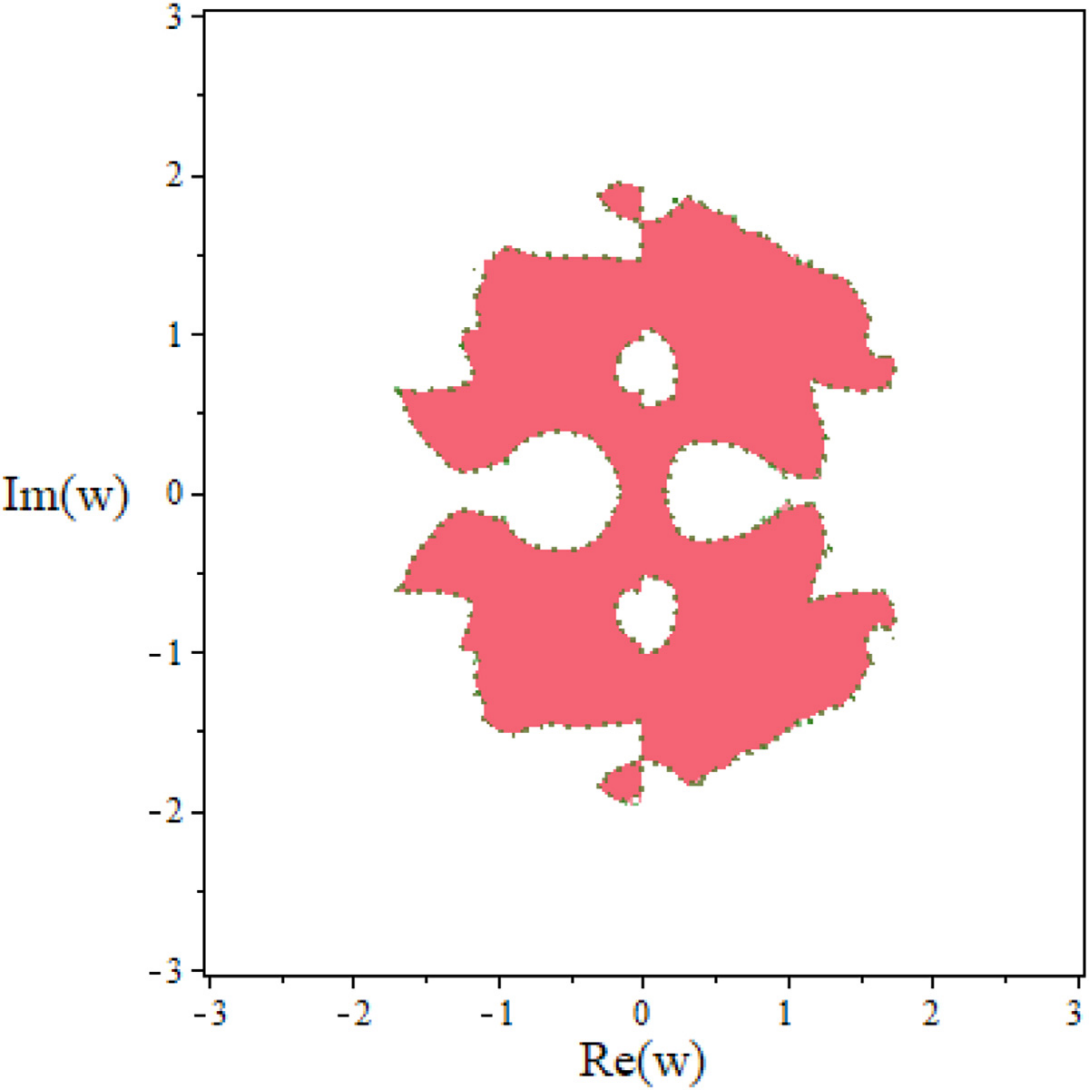
The stability region of corrector for TF-TDMC method.

Using the similar way, we determinant of first characteristic polynomial and set w=0. Stability polynomial is similar as Eq. (32), the roots of stability polynomial are 0,0,1,1. All the roots have modulus less or equal to one as well. It means that the corrector formulae of TF-TDMC method is zero stable. Both predictor and corrector method of TF-TDMC method are zero-stable. The stability region of corrector for TF-TDMC method is shown in Fig. 2.

### Numerical tests and results

In this section, we apply the TF-TDMC method to solve second-order ordinary differential equations of the form $y^{\prime \prime }\left(x\right)=f\left(x,y(x)\right)$ and an application problem featuring a periodic solution. The efficiency of the proposed method in the literature is demonstrated by comparing it with various existing linear multistep methods, including classical-type and fitted techniques. The following methods have been selected for numerical comparison.

• TF-TDMC - Trigonometrically-fitted two derivative linear multistep method in predictor-corrector mode with fifth-order developed in this paper.
• TF-BMCA - Trigonometrically-fitted extra derivative block multistep collocation method in predictor-corrector mode, developed by [21].
• TF-BMCM - Trigonometrically-fitted block multistep methods in predictor-corrector mode with five step number, k=5, developed by [18].

For each second-order oscillatory initial value problem (IVP), a specific fitted frequency, θ can be identified from the analytical solutions. The value of w=θh, where h is the step size, will be determined and substituted into the frequency-dependent parameters for all selected trigonometrically-fitted methods, including the suggested method. Four numerical problems are selected, encompassing some application problems, the Stiefel and Bettis oscillatory problem and two-body problem. Three selected methods, including the proposed method are used to solve all the problems with varying step sizes and endpoints, b. Then, the numerical approximations generated by all selected methods will be compared with the analytical solutions to calculate the maximum global error. Below are the operation steps for TF-TDMC method to outlook the procedures of solving second-order oscillatory IVPs.

### Operation steps of TF-TDMC method

Step 1 Initialization

Start with an initial value for the dependent variable and its derivative, along with the initial conditions and step size, h for the problem. Identify the frequency, θ of the problem and subsequently, determine the frequency-dependent parameters to be used in the predictor and corrector steps of the TF-TDMC method with the information of w=θh.

Step 2 Predictor Step

Use two-step predictor, based on previous values, to estimate the dependent variable, $y_{n+k}$ and its derivative, ${y^{\prime }}_{n+k}$, $k=\frac{5}{3},2$.

Step 3 Corrector Step

Use the predicted values in a corrector formula to refine the estimates of. $y_{n+2}$ and ${y^{\prime }}_{n+2}$ with tolerance of $10^{-20}$. Check for convergence by comparing the predictor and corrector estimates, or by comparing the previous term with the current term in the corrector step. If they are sufficiently close, proceed; if not, iterate until convergence is achieved.

Step 4 Iteration

Repeat the predictor and corrector steps for subsequent time steps, using the updated values from the corrector step as the starting point for the next iteration until reach to the endpoints.

Step 5 Output

Once the desired accuracy is achieved, output the values of the dependent variable at the specified time steps.

### Numerical problems

Problem 1 Homogeneous linear problem

(39) $$\begin{array}{ccc} & & y^{\prime \prime }\left(x\right)=-9y\left(x\right),\\ & & y\left(0\right)=1,\;\;y^{\prime }\left(0\right)=2,\;\;x\in \left[0,b\right], \end{array}$$

with analytical solution, $y\left(x\right)=\frac{2}{3}\sin\left(3x\right)+\cos\left(3x\right).$

The fitted frequency, θ=3.

Problem 2 Homogeneous nonlinear problem

(40) $$\begin{array}{ccc} & & y^{\prime \prime }\left(x\right)=-100y\left(x\right)+\frac{1}{2}\cos\left(x\right)-\sin^{2}\left(x\right),\\ & & y\left(0\right)=1,\;\;y^{\prime }\left(0\right)=1,\;\;x\in \left[0,b\right], \end{array}$$

with analytical solution, $y\left(x\right)=\frac{1}{10}\sin\left(10x\right)+\frac{78767}{79200}\cos\left(10x\right)+\frac{1}{48}\cos^{2}\left(x\right)+\frac{1}{198}\cos\left(x\right)-\frac{49}{2400}.$

The fitted frequency, θ=10.

Problem 3 Stiefel and Bettis oscillatory problem, investigated by [22]

(41) $$\begin{array}{ccc} & & {y^{\prime \prime }}_{1}\left(x\right)=-y_{1}\left(x\right)+0.001\cos\left(x\right),\;{y^{\prime \prime }}_{2}\left(x\right)=-y_{2}\left(x\right)+0.001\sin\left(x\right),\\ & & y_{1}\left(0\right)=1,\;\;{y^{\prime }}_{1}\left(0\right)=0,\;\;y_{2}\left(0\right)=0,\;\;\;{y^{\prime }}_{2}\left(0\right)=0.9995,\;\;x\in \left[0,b\right], \end{array}$$

with analytical solution, $y_{1}\left(x\right)=\cos\left(x\right)+0.0005x\sin\left(x\right)$ and $y_{2}\left(x\right)=\sin\left(x\right)-0.0005x\cos\left(x\right)$.

The fitted frequency, θ=1.

Problem 4 Two-body problem with nonlinear orbital property, investigated by [16]

(42) $$\begin{array}{ccc} & & {y^{\prime \prime }}_{1}\left(x\right)=\frac{-y_{1}\left(x\right)}{{\left[y_{1}^{2}\left(x\right)+y_{2}^{2}\left(x\right)\right]}^{\frac{3}{2}}},\;{y^{\prime \prime }}_{2}\left(x\right)=\frac{-y_{2}\left(x\right)}{{\left[y_{1}^{2}\left(x\right)+y_{2}^{2}\left(x\right)\right]}^{\frac{3}{2}}},\\ & & y_{1}\left(0\right)=1,\;\;{y^{\prime }}_{1}\left(0\right)=0,\;\;y_{2}\left(0\right)=0,\;\;\;{y^{\prime }}_{2}\left(0\right)=1,\;\;x\in \left[0,b\right], \end{array}$$

with analytical solution, $y_{1}\left(x\right)=\cos\left(x\right)$ and $y_{2}\left(x\right)=\sin\left(x\right).$.

The fitted frequency, θ=1.

Problem 5 Nonlinear perturbed Kepler problem with orbital property, studied by [23]

The nonlinear perturbed Kepler problem is a variation of the classical Kepler problem, which is central to celestial mechanics and describes the motion of two bodies under mutual gravitational attraction. This problem is foundational in understanding the orbits of planets, moons, and satellites. The perturbed Kepler problem, particularly the nonlinear version studied by [23], incorporates additional forces or effects that deviate from the simple two-body problem, making the equations of motion more complex and realistic for certain astrophysical scenarios.

### Historical background and derivation

The classical Kepler problem is based on Newton's laws of motion and universal gravitation, yielding elliptical orbits for celestial bodies in a two-body system. The problem is well-known for its simplicity and accuracy in describing planetary motion. However, real celestial bodies are often influenced by other factors, such as additional gravitational forces, relativistic effects, or even the presence of other celestial bodies, leading to deviations from the ideal Keplerian motion. These deviations or perturbations give rise to the perturbed Kepler problem.

In the nonlinear perturbed Kepler problem studied by [23], the differential equations incorporate an additional perturbation term involving a small parameter, ε, which modifies the standard inverse-square law of attraction. The equations describe a more complex interaction that accounts for the perturbative effects on the orbit of a body. To derive the formula for the nonlinear perturbed Kepler problem, we start from the classical Kepler problem and introduce the perturbative term. The classical Kepler problem describes the motion of a body in an inverse-square law gravitational field. The governing differential equation is given by:

(43) $$r^{\prime \prime }\left(t\right)=-\frac{GMr\left(t\right)}{{\left|r\left(t\right)\right|}^{3}},$$

where r(t) is the position vector of the body as a function of time, G is the gravitational constant and M is the mass of the central body. Assume $r\left(t\right)=\left(y_{1}\left(t\right),y_{2}\left(t\right)\right)$ in Cartesian coordinates, where $y_{1}\left(t\right)$ and $y_{2}\left(t\right)$ are the components of the position vector. The equation of motion in Cartesian form is:

(44) $$\begin{array}{ccc} & & {y^{\prime \prime }}_{1}\left(t\right)=-\frac{GMy_{1}\left(t\right)}{{\left[y_{1}^{2}\left(t\right)+y_{2}^{2}\left(t\right)\right]}^{3/2}},\\ & & {y^{\prime \prime }}_{2}\left(t\right)=-\frac{GMy_{2}\left(t\right)}{{\left[y_{1}^{2}\left(t\right)+y_{2}^{2}\left(t\right)\right]}^{3/2}}. \end{array}$$

To account for the perturbation, we add a small perturbative termε to the classical equations. The perturbative term is typically of higher order in the distance, representing additional forces that deviate from the simple inverse-square law. The modified equations become:

(45) $$\begin{array}{ccc} & & {y^{\prime \prime }}_{1}\left(t\right)=-\frac{GMy_{1}\left(t\right)}{{\left[y_{1}^{2}\left(t\right)+y_{2}^{2}\left(t\right)\right]}^{3/2}}-\frac{A\left(\varepsilon \right)y_{1}\left(t\right)}{{\left[y_{1}^{2}\left(t\right)+y_{2}^{2}\left(t\right)\right]}^{5/2}},\\ & & {y^{\prime \prime }}_{2}\left(t\right)=-\frac{GMy_{2}\left(t\right)}{{\left[y_{1}^{2}\left(t\right)+y_{2}^{2}\left(t\right)\right]}^{3/2}}-\frac{A\left(\varepsilon \right)y_{2}\left(t\right)}{{\left[y_{1}^{2}\left(t\right)+y_{2}^{2}\left(t\right)\right]}^{5/2}}. \end{array}$$

where A(ε) is a function of the perturbation parameter ε.

**Fig. 3:**
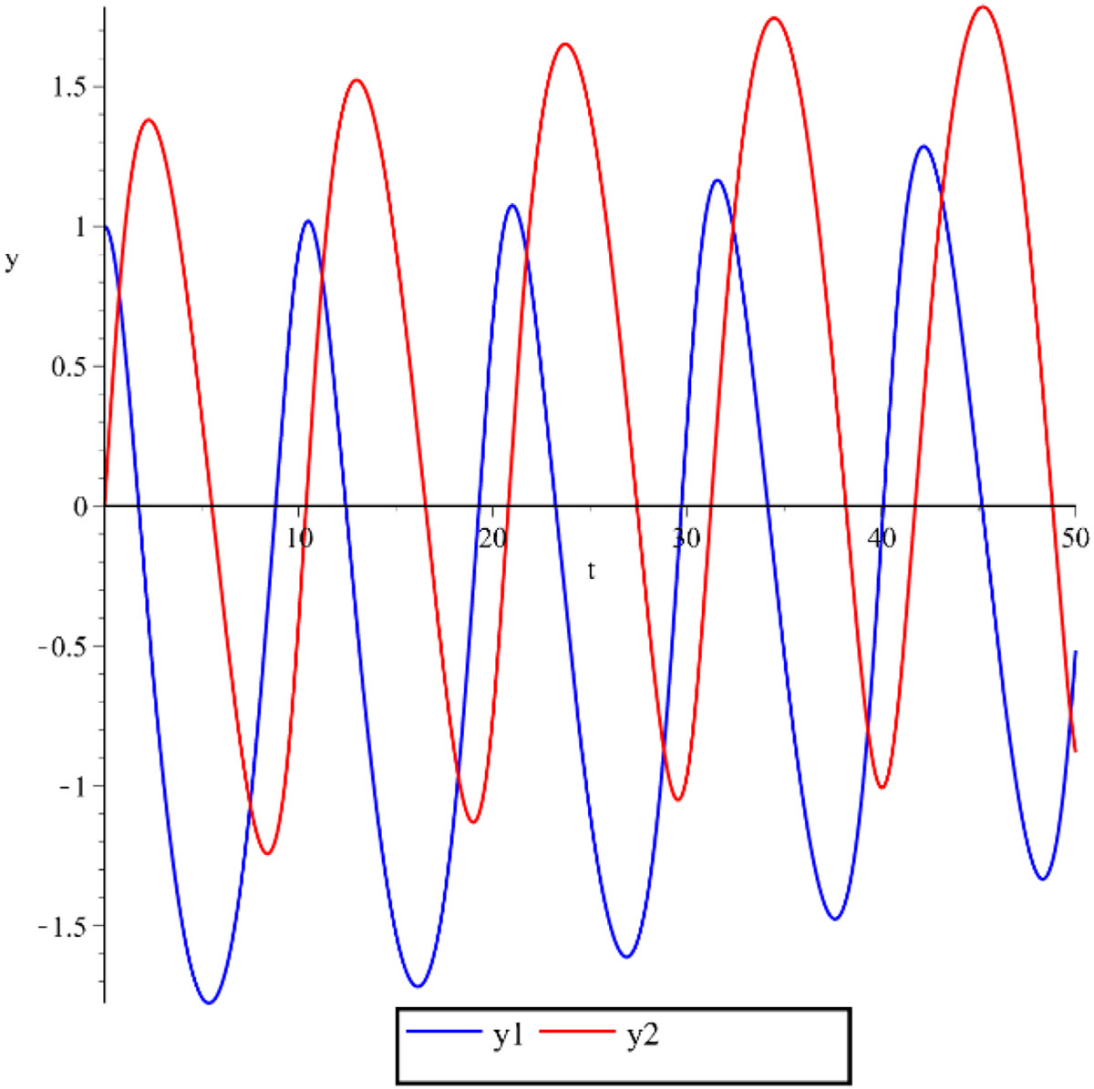
Numerical simulation for the perturbed Kepler problem, $GM=1,\;A\left(\varepsilon \right)=\varepsilon^{2}-\varepsilon ,\;y_{1}\left(0\right)=1,\;y_{2}\left(0\right)=0,\;$${y^{\prime }}_{1}\left(0\right)=0,\;{y^{\prime }}_{2}\left(0\right)=1+\varepsilon ,\;\varepsilon =0.1$ and t∈[0,50].

**Fig. 4:**
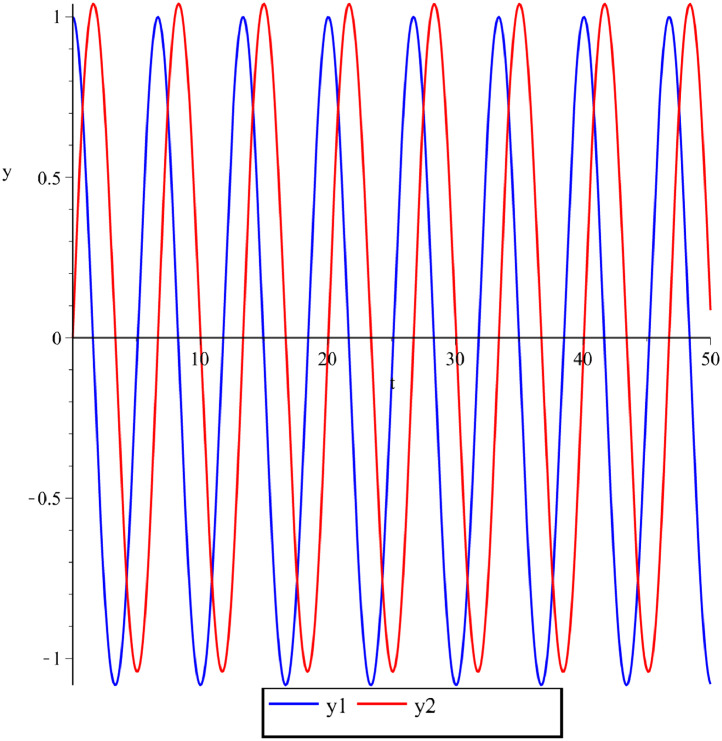
Numerical simulation for the perturbed Kepler problem, $GM=1,\;A\left(\varepsilon \right)=\varepsilon^{2}-\varepsilon^{3},\;y_{1}\left(0\right)=1,\;y_{2}\left(0\right)=0,\;$${y^{\prime }}_{1}\left(0\right)=0,\;{y^{\prime }}_{2}\left(0\right)=1+\varepsilon ,\;\varepsilon =0.02$ and t∈[0,50].

**Fig. 5:**
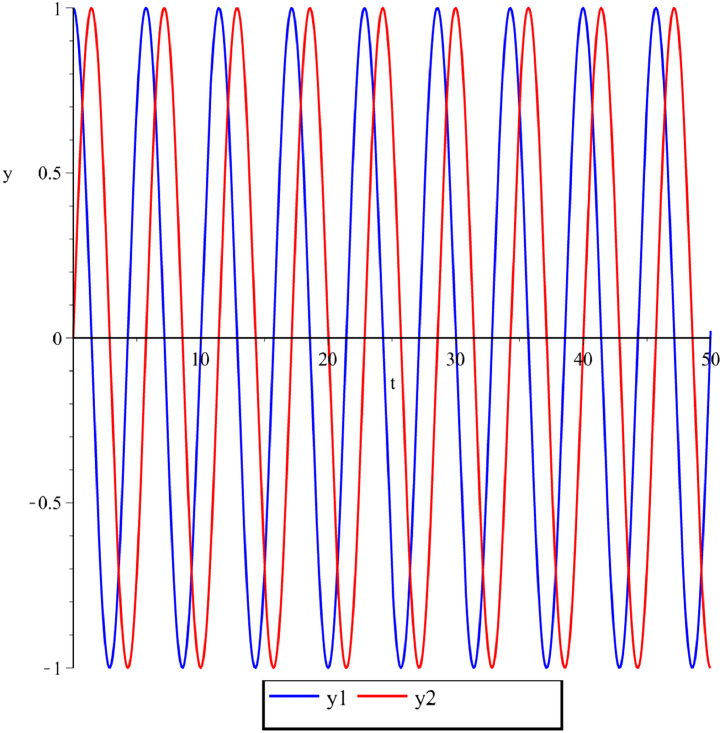
Numerical simulation for the perturbed Kepler problem, $GM=1,\;A\left(\varepsilon \right)=2\varepsilon +\varepsilon^{2},\;y_{1}\left(0\right)=1,\;y_{2}\left(0\right)=0,\;$${y^{\prime }}_{1}\left(0\right)=0,\;{y^{\prime }}_{2}\left(0\right)=1+\varepsilon ,\;\varepsilon =0.1$ and t∈[0,50].

Below are some numerical simulations with different parameters and are displayed in Fig. 3, Fig. 4, Fig. 5 using classical Runge-Kutta method with $h=10^{-5}.$

In the study presented by [23], $GM=1,\;A\left(\varepsilon \right)=2\varepsilon +\varepsilon^{2}$ and set x as independent variable, the nonlinear perturbed Kepler problem with orbital properties can be simplified into:

(46) $$y_{1}^{\prime \prime }\left(x\right)=-\frac{y_{1}\left(x\right)}{{\left[y_{1}^{2}\left(x\right)+y_{2}^{2}\left(x\right)\right]}^{3/2}}-\frac{\left(2\varepsilon +\varepsilon^{2}\right)y_{1}\left(x\right)}{{\left[y_{1}^{2}\left(x\right)+y_{2}^{2}\left(x\right)\right]}^{5/2}},\;y_{1}^{\prime \prime }\left(x\right)=-\frac{y_{2}\left(x\right)}{{\left[y_{1}^{2}\left(x\right)+y_{2}^{2}\left(x\right)\right]}^{3/2}}-\frac{\left(2\varepsilon +\varepsilon^{2}\right)y_{2}\left(x\right)}{{\left[y_{1}^{2}\left(x\right)+y_{2}^{2}\left(x\right)\right]}^{5/2}}.$$

with the initial conditions: $y_{1}\left(0\right)=1,\;\;{y^{\prime }}_{1}\left(0\right)=0,\;\;y_{2}\left(0\right)=0,\;\;{y^{\prime }}_{2}\left(0\right)=1+\varepsilon .$

The exact solution is given by$y_{1}\left(x\right)=\cos\left[(1+\varepsilon )x\right]$ and$y_{2}\left(x\right)=\sin\left[(1+\varepsilon )x\right]$ with fitted frequency, θ=1+ε. In this study, we use a perturbed value of ε=0.001.

Table 1, Table 2, Table 3, Table 4, Table 5 demonstrate the numerical performance of proposed method and other selected methods in term of maximum global truncation error (ERROR) against computational time in seconds (TIME). The model of computer used in computing the numerical results is Lenovo ideapad 330 Intel Core i5–8050 U (1.8 GHz). The software utilized for computation is Maplesoft 2023, a mathematical tool known for its user-friendly interface that facilitates easy analysis, visualization, and exploration of mathematical concepts.

**Table 1:** Numerical comparison between TF-TDMC method with existing methods for problem 1.

h	METHODS		b=100		b=1000	
			ERROR	TIME	ERROR	TIME
0.1	TF-TDMC		1.864776 × 10^–12^	0.154	1.891792 × 10^–11^	2.407
	TF-BMCA		1.516316 × 10^–5^	0.170	1.533537 × 10^–5^	2.565
	TF-BMCM		1.985112 × 10^–6^	0.135	2.003516 × 10^–5^	2.101
0.05	TF-TDMC		4.387876 × 10^–16^	0.282	4.415057 × 10^–15^	4.795
	TF-BMCA		2.118361 × 10^–7^	0.317	2.373446 × 10^–7^	4.981
	TF-BMCM		1.861337 × 10^–9^	0.245	1.874340 × 10^–8^	4.230
0.025	TF-TDMC		1.059278 × 10^–19^	0.556	1.066258 × 10^–18^	8.858
	TF-BMCA		2.493108 × 10^–9^	0.632	3.701041 × 10^–9^	9.863
	TF-BMCM		1.801416 × 10^–12^	0.456	1.812594 × 10^–11^	7.476
0.0125	TF-TDMC		2.578761 × 10^–23^	1.142	2.596275 × 10^–22^	17.459
	TF-BMCA		2.477719 × 10^–11^	1.345	5.759004 × 10^–11^	18.007
	TF-BMCM		1.768468 × 10^–15^	0.927	1.766190 × 10^–14^	15.151
0.00625	TF-TDMC		6.292172 × 10^–27^	2.244	6.334969 × 10^–26^	33.209
	TF-BMCA		2.204758 × 10^–13^	2.623	8.486645 × 10^–13^	33.423
	TF-BMCM		1.727434 × 10^–18^	1.837	1.724193 × 10^–17^	27.830

**Table 2:** Numerical comparison between TF-TDMC method with existing methods for problem 2.

h	METHODS	b=100		b=1000	
		ERROR	TIME	ERROR	TIME
0.06	TF-TDMC	3.692032 × 10^–7^	2.036	4.555166 × 10^–7^	14.545
	TF-BMCA	9.224050 × 10^–4^	2.149	9.225949 × 10^–4^	16.678
	TF-BMCM	7.744874 × 10^–3^	1.896	8.098014 × 10^–2^	12.678
0.05	TF-TDMC	1.726065 × 10^–7^	2.633	1.726065 × 10^–7^	19.253
	TF-BMCA	3.271016 × 10^–4^	2.866	3.277727 × 10^–4^	22.520
	TF-BMCM	1.072426 × 10^–3^	2.337	1.081199 × 10^–2^	17.053
0.04	TF-TDMC	6.902935 × 10^–8^	3.160	6.902935 × 10^–8^	24.124
	TF-BMCA	1.090319 × 10^–4^	3.310	1.090350 × 10^–4^	27.568
	TF-BMCM	1.044428 × 10^–4^	2.818	1.046306 × 10^–3^	21.820
0.03	TF-TDMC	2.144751 × 10^–8^	3.862	2.144751 × 10^–8^	29.471
	TF-BMCA	4.014011 × 10^–5^	4.029	4.014052 × 10^–5^	33.542
	TF-BMCM	5.536977 × 10^–6^	3.339	5.557081 × 10^–5^	26.534
0.02	TF-TDMC	4.184704 × 10^–9^	5.556	4.184843 × 10^–9^	37.785
	TF-BMCA	1.937355 × 10^–5^	5.904	1.937405 × 10^–5^	42.153
	TF-BMCM	9.296420 × 10^–8^	5.298	9.322769 × 10^–7^	32.543

**Table 3:** Numerical comparison between TF-TDMC method with existing methods for problem 3.

h	METHODS	b=10		b=100	
		ERROR	TIME	ERROR	TIME
0.125	TF-TDMC	1.330810 × 10^–8^	0.035	1.396892 × 10^–7^	0.341
	TF-BMCA	3.450393 × 10^–8^	0.039	2.678761 × 10^–7^	0.381
	TF-BMCM	2.526971 × 10^–7^	0.033	4.805749 × 10^–2^	0.300
0.10	TF-TDMC	2.628444 × 10^–9^	0.051	2.761409 × 10^–8^	0.530
	TF-BMCA	6.453101 × 10^–9^	0.058	5.471769 × 10^–8^	0.586
	TF-BMCM	3.403212 × 10^–8^	0.048	8.066130 × 10^–6^	0.480
0.075	TF-TDMC	8.324668 × 10^–10^	0.071	8.739164 × 10^–9^	0.782
	TF-BMCA	2.040122 × 10^–9^	0.058	1.821294 × 10^–8^	0.820
	TF-BMCM	8.126340 × 10^–9^	0.048	1.363590 × 10^–7^	0.745
0.05	TF-TDMC	1.644482 × 10^–10^	0.084	1.726436× 10^–9^	0.952
	TF-BMCA	4.045599 × 10^–10^	0.099	3.807051 × 10^–9^	1.000
	TF-BMCM	1.075513 × 10^–9^	0.079	1.144815 × 10^–8^	0.874
0.025	TF-TDMC	1.027859 × 10^–11^	0.175	1.079250 × 10^–10^	1.868
	TF-BMCA	2.542065 × 10^–11^	0.196	2.525066 × 10^–10^	1.982
	TF-BMCM	3.371494 × 10^–11^	0.166	3.538982 × 10^–10^	1.615

**Table 4:** Numerical comparison between TF-TDMC method with existing methods for problem 4.

h	METHODS	b=10		b=100	
		ERROR	TIME	ERROR	TIME
0.1	TF-TDMC	1.353704 × 10^–19^	0.066	3.406336 × 10^–16^	0.802
	TF-BMCA	2.938062 × 10^–8^	0.072	3.176734 × 10^–4^	0.962
	TF-BMCM	1.092689 × 10^–16^	0.061	3.402128 × 10^–16^	0.645
0.05	TF-TDMC	4.528368 × 10^–23^	0.135	1.376001 × 10^–21^	1.509
	TF-BMCA	1.981663 × 10^–10^	0.142	1.688161 × 10^–7^	1.789
	TF-BMCM	2.763311 × 10^–20^	0.119	2.634101 × 10^–19^	1.257
0.025	TF-TDMC	1.540465 × 10^–26^	0.265	1.027510 × 10^–25^	2.585
	TF-BMCA	1.433911 × 10^–12^	0.286	4.168009 × 10^–10^	3.268
	TF-BMCM	6.788675 × 10^–24^	0.242	7.156790 × 10^–23^	2.254
0.0125	TF-TDMC	4.406450 × 10^–30^	0.546	1.060644 × 10^–29^	5.736
	TF-BMCA	1.077299 × 10^–14^	0.607	1.944607 × 10^–12^	7.326
	TF-BMCM	1.664261 × 10^–27^	0.519	1.765264 × 10^–26^	5.012
0.00625	TF-TDMC	1.161969 × 10^–33^	1.120	4.955544 × 10^–33^	13.278
	TF-BMCA	8.252017 × 10^–17^	1.256	1.192627 × 10^–14^	15.507
	TF-BMCM	4.074683 × 10^–31^	1.038	4.313005 × 10^–30^	11.121

**Table 5:** Numerical comparison between TF-TDMC method with existing methods for problem 5.

h	METHODS	b=10		b=100	
		ERROR	TIME	ERROR	TIME
0.1	TF-TDMC	1.654299 × 10^–19^	0.095	2.077801 × 10^–17^	0.981
	TF-BMCA	2.504817 × 10^–8^	0.126	3.201751 × 10^–6^	1.322
	TF-BMCM	1.107823 × 10^–16^	0.072	3.433128 × 10^–16^	0.841
0.05	TF-TDMC	6.122643 × 10^–23^	0.192	2.077882 × 10^–21^	2.003
	TF-BMCA	1.837986 × 10^–10^	0.250	3.588304 × 10^–8^	2.621
	TF-BMCM	2.801710 × 10^–20^	0.149	2.672955 × 10^–19^	1.735
0.025	TF-TDMC	1.813528 × 10^–26^	0.389	1.410970 × 10^–25^	4.010
	TF-BMCA	1.388878 × 10^–12^	0.506	2.247937 × 10^–10^	5.214
	TF-BMCM	6.884213 × 10^–24^	0.303	7.269413 × 10^–23^	3.370
0.0125	TF-TDMC	4.816545 × 10^–30^	0.756	1.712940 × 10^–29^	7.944
	TF-BMCA	1.066655 × 10^–14^	1.001	1.488749 × 10^–12^	10.345
	TF-BMCM	1.687701 × 10^–27^	0.609	1.793263 × 10^–26^	6.590
0.00625	TF-TDMC	1.223413 × 10^–33^	1.531	7.969373 × 10^–33^	15.738
	TF-BMCA	8.261070 × 10^–17^	1.997	1.058971 × 10^–14^	20.459
	TF-BMCM	4.132141 × 10^–31^	1.265	4.381653 × 10^–30^	13.089

**Fig. 6:**
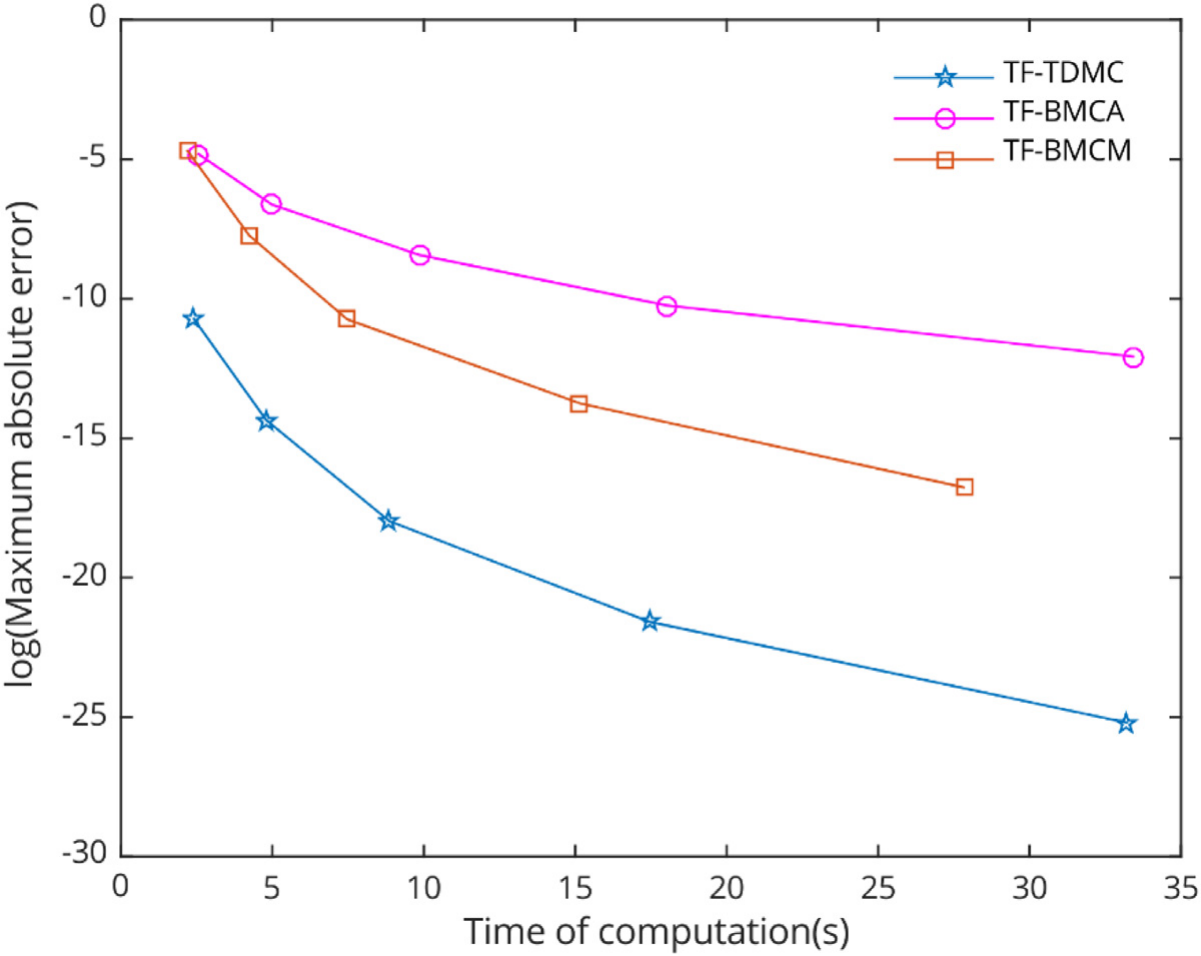
Numerical efficiency of selected methods for problem 1 with b=1000 and $h=\frac{0.1}{2^{i}},\;\;i=0,1,...,4.$.

**Fig. 7:**
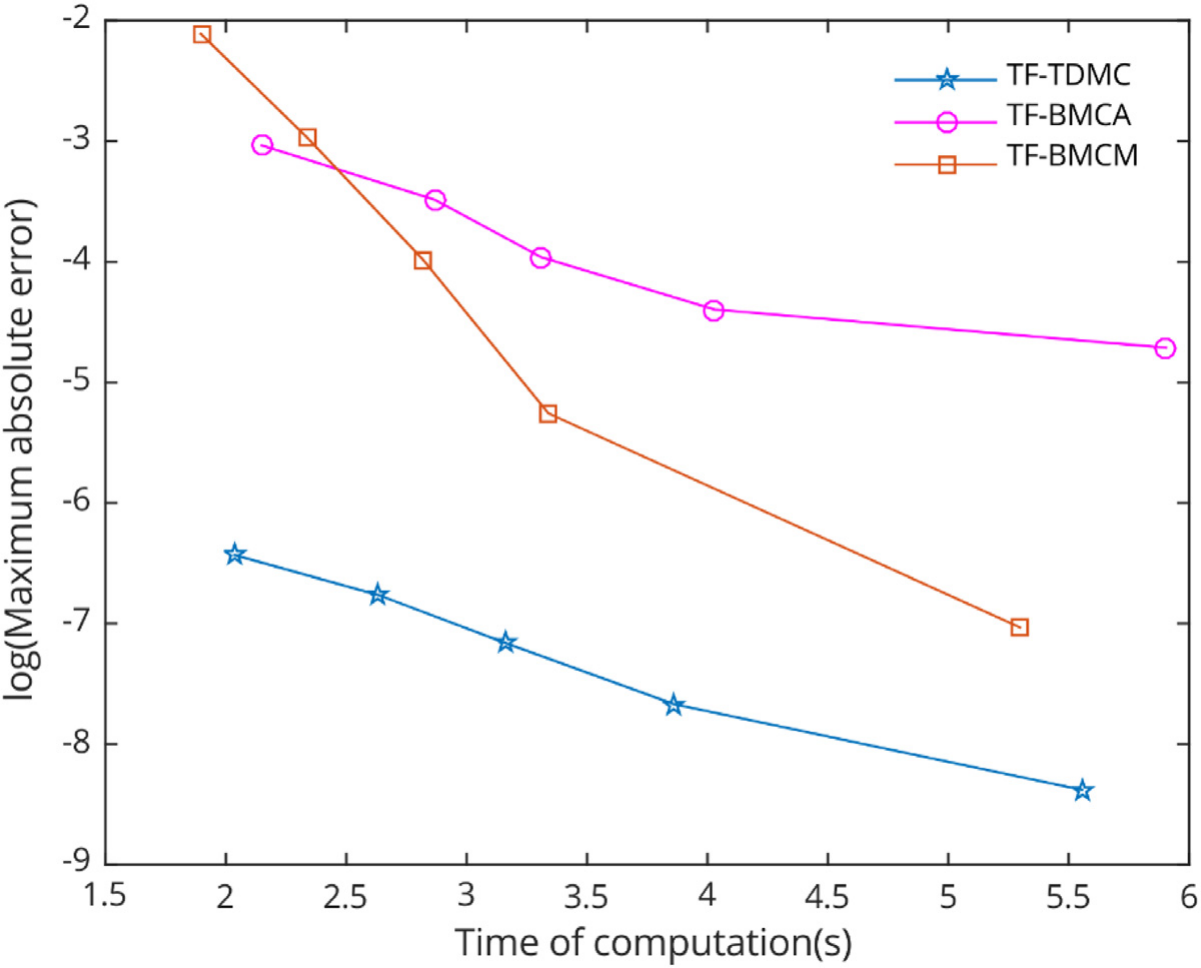
Numerical efficiency of selected methods for problem 2 with b=100 and h=0.06−0.01i,i=0,1,...,4..

**Fig. 8:**
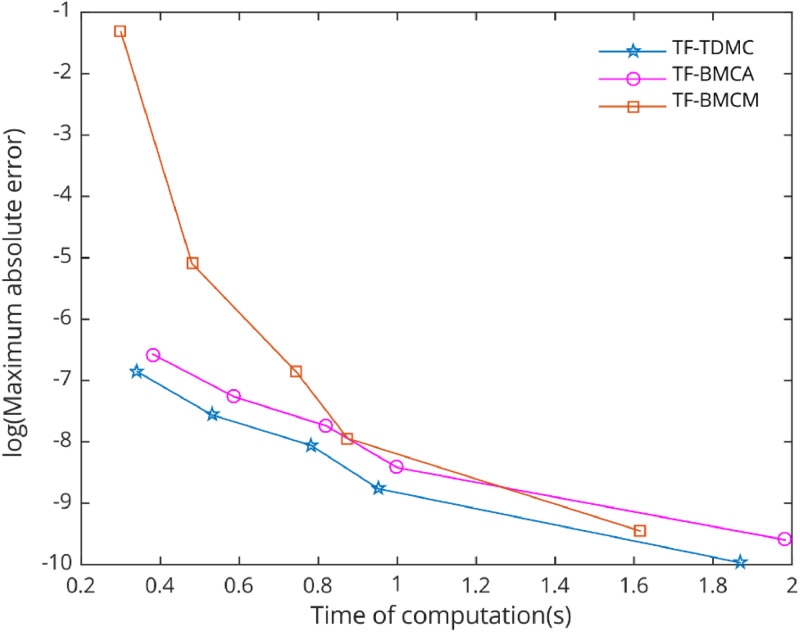
Numerical efficiency of selected methods for problem 3 with b=50 and h=0.125−0.025i,i=0,1,...,4..

**Fig. 9:**
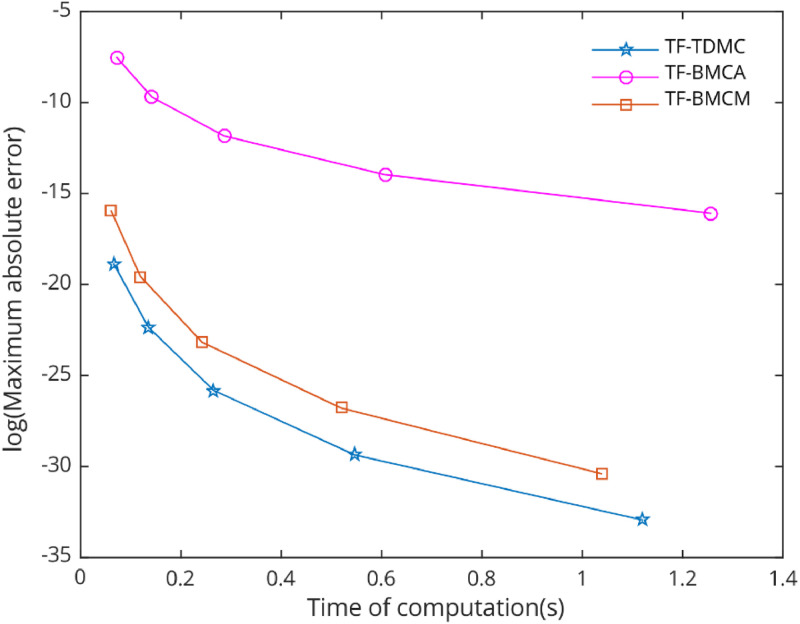
Numerical efficiency of selected methods for problem 4 with b=10 and $h=\frac{0.1}{2^{i}},\;\;i=0,1,...,4.$.

**Fig. 10:**
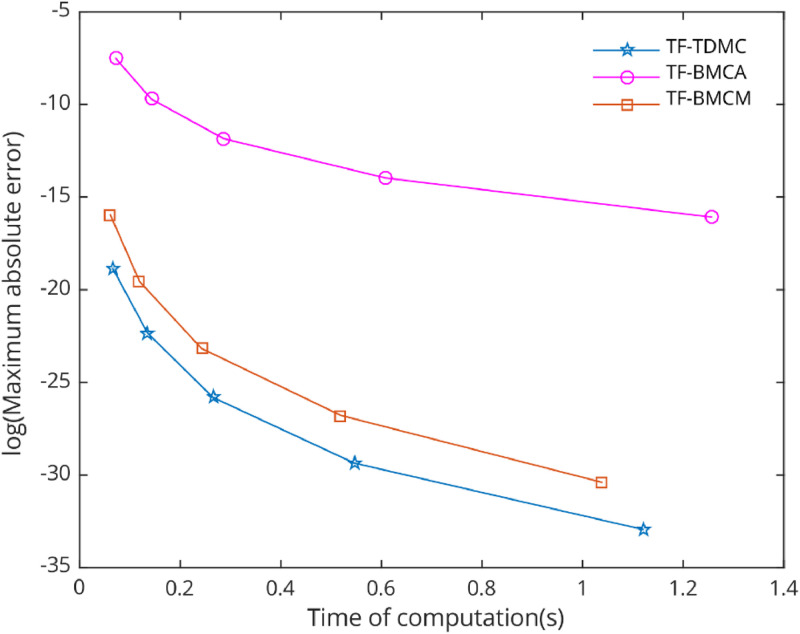
Numerical efficiency of selected methods for problem 5 with b=10 and $h=\frac{0.1}{2^{i}},\;\;i=0,1,...,4.$.

Fig. 6, Fig. 7, Fig. 8, Fig. 9, Fig. 10 demonstrate the numerical performance of proposed method and other selected methods in term of maximum global truncation error against computational time.

## Discussion and conclusion

In the numerical test, five types of problems with periodic solutions were selected to evaluate the performance of the chosen methods. The TF-TDMC method was compared to existing trigonometric-fitted block multistep methods in predictor-corrector mode. The evaluation focused on computation time and maximum global error produced by each method, with results presented in Table 1, Table 2, Table 3, Table 4, Table 5 and Fig. 6, Fig. 7, Fig. 8, Fig. 9, Fig. 10. The inclusion of the two-derivative term significantly enhances accuracy, especially when combined with the trigonometric-fitting technique. This combination minimizes error at every stage, particularly with sufficiently small step sizes and these benefits lead to TF-TDMC method produces the smallest maximum global error among all methods across different step sizes and endpoints, demonstrating its superior performance. The TF-TDMC method has a distinct advantage over other methods when solving problems involving only trigonometric functions, such as problems 1 and 4. However, in the case of problems 2 and 3, where the exact solutions involve nonlinearities and non-trigonometric functions, the difference in global truncation error between the TF-TDMC method and other methods, such as the TF-BMCM, was smaller. This suggests that while the TF-TDMC method excels in solving problems with trigonometric solutions, its performance may be slightly less pronounced when dealing with solutions that incorporate non-trigonometric components. The presence of these components can introduce inaccuracies in the fitting process, leading to relatively higher global errors compared to purely oscillatory cases.

In the derivation of the TF-TDMC method, the extra derivative plays a crucial role in enhancing numerical performance. By utilizing additional derivative information, the numerical approximation can more accurately reflect the actual behavior of the solution, particularly over larger intervals. This leads to reduced local truncation errors. In many numerical methods, especially those addressing differential equations, errors can accumulate over iterations. The inclusion of an extra derivative allows for a better representation of the solution's curvature and dynamic behavior, helping to compensate for potential errors introduced in earlier steps and thereby improving overall accuracy.

The derivation of the multistep method is motivated by its ability to achieve higher-order accuracy compared to one-step methods. By utilizing multiple previous points in the solution, multistep collocation methods can offer a more precise approximation, particularly for problems that require fine resolution. Additionally, stiff differential equations pose challenges for one-step methods, which often necessitate small time steps for stability. In contrast, multistep methods can manage stiffness more effectively, allowing for larger time steps while maintaining both stability and accuracy. Furthermore, by considering multiple points, multistep collocation methods enhance interpolation between known values, resulting in a smoother approximation of the solution that is especially beneficial for capturing complex behaviors.

Regarding the rationale for choosing collocation points, we select relatively small distances between them to achieve more precise approximations of the solution. By sampling the function more frequently, the method can capture finer details and variations, resulting in reduced approximation errors. When collocation points are closer together, the method tends to exhibit improved numerical stability, which is particularly crucial for stiff equations where small perturbations can cause significant changes in the solution. This close placement of points helps maintain stability throughout the iterations.

The trigonometrically-fitting technique is instrumental in aligning the numerical method with the exact solution's oscillatory nature, thereby significantly reducing phase and amplitude errors. This alignment ensures that the TF-TDMC method remains stable across a wide range of step sizes, preventing the numerical solution from diverging or exhibiting unphysical behavior. As a result, the TF-TDMC method proves to be highly efficient and reliable for solving second-order ODEs with oscillatory solutions, making it a valuable addition to the existing toolbox of numerical methods for such problems.

## Limitations

1. TF-TDMC method cannot be used to solve general second-order ordinary differential equations in the form of $y^{\prime \prime }=f\left(x,y,y^{\prime }\right)$. This is because the method specially designed to solve $y^{\prime \prime }=f\left(x,y\right)$. The derivation of method is based on $y^{\prime \prime }=f\left(x,y\right)$, including the f function and extra derivative, g function.
2. TF-TDMC method is less effective when solving $y^{\prime \prime }=f\left(x,y\right)$ with non-trigonometric solutions. This is because the implementation of the trigonometric fitting technique aims to effectively address problems with periodic solutions. For problems that do not have trigonometric solutions, the numerical performance of the TF-TDMC method is nearly comparable to that of other classical two-derivative multistep collocation methods, no much difference in term of absolute maximum global error.
3. For large systems of ordinary differential equations or stiff equations, the computation using Maplesoft can be slow or may require significant memory.

## CRediT author statement

**Khai Chien Lee**: Conceptualization, Methodology, Software, Formal analysis, Investigation, Resources, Data Curation, Writing - Original Draft, Visualization, Project administration, Funding acquisition. **Muhammad Naeim Mohd Aris**: Methodology, Validation, Investigation, Writing - Review & Editing. **Ishak Hashim**: Validation, Formal analysis, Writing - Review & Editing, Supervision, Project administration. **Norazak Senu**: Validation, Writing - Review & Editing, Supervision, Project administration.

## Ethics statements

Not applicable.

## Declaration of competing interest

The authors declare that they have no known competing financial interests or personal relationships that could have appeared to influence the work reported in this paper.

## Data Availability

Data will be made available on request.
